# Cognitive impairment in diffuse axonal injury patients with favorable outcome

**DOI:** 10.3389/fnins.2023.1077858

**Published:** 2023-01-25

**Authors:** Weiliang Chen, Chunyu Yao, Shengwen Li, Hongguang Huang, Zujian Zhu, Rui Chen, Wen Su, Xiao Huang, Lisheng Xu, Kaijie Sun, Jiannan Song, Rongcai Jiang, Guanjun Wang

**Affiliations:** ^1^Department of Neurosurgery, Haining People’s Hospital, Jiaxing, Zhejiang, China; ^2^The Second Department of Orthopaedics, Haining People’s Hospital, Haining, Zhejiang, China; ^3^Department of Neurosurgery, The First Affiliated Hospital of Zhejiang University Medical College, Hangzhou, Zhejiang, China; ^4^Department of Neurosurgery, Tianjin Medical University General Hospital, Tianjin, China

**Keywords:** diffuse axonal injury, cognitive impairment, outcome, Montreal cognitive assessment, cognitive domain

## Abstract

**Background and purpose:**

Traumatic brain injury (TBI), especially the severe TBI are often followed by persistent cognitive sequalae, including decision-making difficulties, reduced neural processing speed and memory deficits. Diffuse axonal injury (DAI) is classified as one of the severe types of TBI. Part of DAI patients are marginalized from social life due to cognitive impairment, even if they are rated as favorable outcome. The purpose of this study was to elucidate the specific type and severity of cognitive impairment in DAI patients with favorable outcome.

**Methods:**

The neurocognition of 46 DAI patients with favorable outcome was evaluated by the Chinese version of the Montreal Cognitive Assessment Basic (MoCA-BC), and the differences in the domains of cognitive impairment caused by different grades of DAI were analyzed after data conversion of scores of nine cognitive domains of MoCA-BC by Pearson correlation analysis.

**Results:**

Among the 46 DAI patients with favorable outcome, eight had normal cognitive function (MoCA-BC ≥ 26), and 38 had cognitive impairment (MoCA-BC < 26). The MoCA-BC scores were positively correlated with pupillary light reflex (*r* = 0.361, *p* = 0.014), admission Glasgow Coma Scale (GCS) (*r* = 0.402, *p* = 0.006), and years of education (*r* = 0.581, *p* < 0.001). Return of consciousness (*r* = −0.753, *p* < 0.001), Marshall CT (*r* = −0.328, *p* = 0.026), age (*r* = −0.654, *p* < 0.001), and DAI grade (*r* = −0.403, *p* = 0.006) were found to be negatively correlated with the MoCA-BC scores. In patients with DAI grade 1, the actually deducted scores (Ads) of memory (*r* = 0.838, *p* < 0.001), abstraction (*r* = 0.843, *p* < 0.001), and calculation (*r* = 0.782, *p* < 0.001) were most related to the Ads of MoCA-BC. The Ads of nine cognitive domains and MoCA-BC were all proved to be correlated, among patients with DAI grade 2. However, In the DAI grade 3 patients, the highest correlation with the Ads of MoCA-BC were the Ads of memory (*r* = 0.904, *p* < 0.001), calculation (*r* = 0.799, *p* = 0.006), orientation (*r* = 0.801, *p* = 0.005), and executive function (*r* = 0.869, *p* = 0.001).

**Conclusion:**

DAI patients with favorable outcome may still be plagued by cognitive impairment, and different grades of DAI cause different domains of cognitive impairment.

## Introduction

DAI is caused by acceleration-deceleration or rotational forces on brain tissues, resulting in axonal shear injuries and delayed axonal disconnection, categorized as a special type of traumatic brain injury (TBI) (Mu et al., 2021; Zhou et al., 2021; Chen et al., 2022; Kim et al., 2022). A total of 20–38% of TBI patients with DAI were followed up with unfavorable outcome (Extended Glasgow Outcome Scale, GOSE 1–4) (Humble et al., 2018; Lohani et al., 2020), but over 85% suffered from persistent cognitive impairment (Macruz et al., 2022; RaukolaLindblom et al., 2022). Significant differences appeared in the incidence of unfavorable outcome and cognitive impairment, reflecting that part of DAI patients classified as favorable outcome were still plagued by cognitive impairment and could not return to normal social life. However, at present, the prevalence and specificity of cognitive impairment in diffuse axonal injury patients with favorable outcome remain unclear.

“Neurocognition” is defined as a collection of interrelated domains such as executive function (EF), language and perceptual motor function, calculation, complex attention, memory, and visuoperception (George et al., 2018; Holguin et al., 2022; RaukolaLindblom et al., 2022). Found that the Finnish KAT test is a valuable tool to detect cognitive-linguistic deficits by comparing the cognitive function of 48 adults with moderate to severe DAI and 27 healthy controls. The Hopkins Language Learning Test (HVLT), Trail Making Test (TMT), and Rey–Osterrieth Complex Figure test were used to assess neuropsychological results in 25 DAI patients at 6 and 12 months post-traumatic, revealed that patients’ episodic verbal memory, attention, and executive function were improved at 6 and 12 months after the trauma (Macruz et al., 2022). All of the above test methods could only evaluate certain domains of Neurocognition, and the Montreal Cognitive Assessment (MoCA) (Nasreddine et al., 2005) was considered to be a more appropriate scale to comprehensively assess cognitive function after traumatic brain injury, subarachnoid hemorrhage, stroke and Alzheimer’s disease (Cornea et al., 2022; Holguin et al., 2022; Wang et al., 2022). MoCA and resting-state perfusion magnetic resonance imaging were performed in 40 patients with acute mild TBI and 40 healthy controls within 14 days following injury to elucidate the relationship between cerebral blood flow connectivity differences and cognitive outcomes in the acute phase after mild TBI (Duan et al., 2022). Further more, a study from Shanghai Huashan Hospital confirmed that the Chinese version of the Montreal Cognitive Assessment Basic (MoCA-BC), as a reliable cognitive screening test across all education levels, has the advantages of high acceptance and good reliability, and is more suitable than MoCA for Chinese elderly adults with low years of education (Chen et al., 2016).

The favorable outcome (GOSE 5–8) can not be used as the final prognostic indicator because a large part of DAI patients is still troubled by cognitive impairment at several months after TBI (George et al., 2018). We hypothesized that DAI patients with favorable outcomes still have cognitive impairment in different domains. To test this hypothesis, we analyzed the functional status of nine cognitive domains in patients with DAI at 6 months after injury.

## Materials and methods

### Participants

Patients with DAI diagnosed by magnetic resonance imaging (MRI) within 30 days after injury were screened in this retrospective observational study between January 2019 and December 2021. Neuropsychological of DAI patients were assessed by MoCA-BC at 6 months after TBI. This data collection site was approved by the local Institutional Review Board, and written informed consent was obtained from all participants or their representatives.

The inclusion criteria were the following: TBI patients’ age between 18 and 70 years, diagnosed as DAI by clinical MRI scan within 30 days after injury, 6-month GOSE score ≥5, years of education ≥4. The exclusion criteria were the following: Progressive brain illness (Dementia, Parkinson disease, multiple sclerosis, seizure disorder, and brain tumor) (2/8), history of brain surgery or stroke without full recovery (1/8), Unable to complete or cooperate with the cognitive function test (speech disorder or mental illness) (5/8).

### Parameters

Demographic and clinical characteristics were collected during hospitalization, including age, sex, causes of trauma (road traffic accident, fall, and others), years of education, admission GCS score, pupillary light reflex (none, unilateral or bilateral), Marshall CT classification (evaluated on a scale from 1 to 6) from the first head CT scan, time of the return of consciousness.

### Diagnosis and grading of DAI

The clinical MRI was performed with a 1.5T scanner (Siemens Symphony, ATim) within 30 days after injury. DAI were defined as TBI patients with lesions in gray-white matter junction of the cerebrum, corpus callosum, or brain stem with T2-weighted imaging (T2WI), T2-weighted fluid attenuated inversion recovery (T2 FLAIR), and diffusion-weighted imaging (DWI) in magnetic resonance imaging (MRI) (Subash et al., 2020; Yue et al., 2020). These lesions were defined as having hypointense focus presented on T2WI (hemorrhagic DAI), or hyperintense focus presented on DWI, T2 FLAIR, and T2WI (non-hemorrhagic DAI) (Rutman et al., 2017; Benjamini et al., 2021). The MRI and CT images were independently analyzed by two experienced neuroradiologists, who had access to patient clinical information but were blinded to the cognitive function.

**FIGURE 1:**
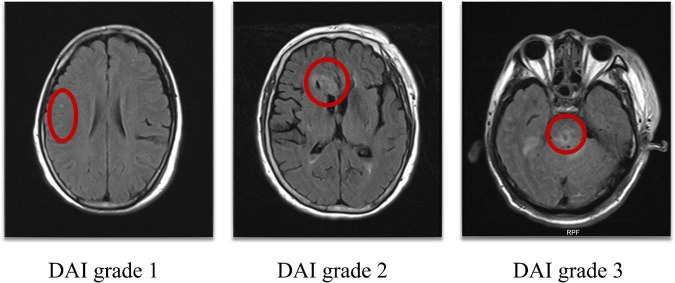
DAI grade 1: Lesions in the hemispheres, DAI grade 2: Lesions in the corpus callosum, DAI grade 3: Lesions in the brainstem (as shown in the red circle). DAI, Diffuse axonal injury.

The presence of DAI in the hemispheres or cerebellum was recorded as DAI grade 1, in the corpus callosum with or without lesions of grade 1 as DAI grade 2, and in the brainstem with or without lesions of grade 1 and/or 2 as DAI grade 3. Patients without DAI were assigned to grade 0 (Adams et al., 1989; Figure 1).

### Extended Glasgow outcome scale

GOSE was used to quantify 6-month outcome as favorable outcome (GOSE 5–8; no or moderate disability) or unfavorable outcome (GOSE 1–4; severe disability or death). GOSE was divided into eight levels (Wilson et al., 1998; Table 1).

**TABLE 1:** Extended Glasgow Outcome Scale.

Unfavorable outcome	Favorable outcome
1: Death	5: Moderately disabled, qualified for low-level work
2: Plant state	6: Moderately disabled, resuming the previous job but requiring some adjustments
3: Severely disabled and totally dependent on others for daily activities	7: Good recovery, and accompanied by minor physical and mental deficits
4: Severely disabled and can do some daily activities with the help of others	8: Great recovery

### Neuropsychological assessment

The MoCA-BC was translated from the original English version with subtle linguistic and cultural modifications and regarded as an effective cognitive test for Chinese elderly adults with low years of education (Chen et al., 2016; Huang et al., 2018). The MoCA-BC assesses nine cognitive domains including executive function, language, orientation, calculation, abstraction, memory, visuoperception, naming, and attention. It takes about 10 min to complete the test, with a maximum score of 30 points. More than 26 points was regarded as normal and with a lower score indicating greater cognitive impairment (Zhang et al., 2019). Each patient’s test was performed by trained staff in a quiet environment at 6 months after injury.

### Data conversion

The scores of nine cognitive domains of MoCA-BC were all converted into the actually deducted scores (Ads), because the study focused on analyzing the negative impact of cognitive function (Rainer et al., 2006). The specific conversion method was to subtract the total score of each cognitive domain from the actual score of this domain, that is, the negative value of the score deducted from each domain, for example, if the patient’s orientation score was four, then his a Ads of orientation was four minus six (the total score of orientation), which equals to negative two points.

### Statistical analysis

All statistical analyses were performed using GraphPad Prism 8 (GraphPad Software, San Diego, CA, USA). A value of *p* < 0.05 with a two-tailed test was considered statistically significant. Categorical data are presented as frequency or percentage and compared by Fisher exact test. Continuous data are presented as the median and interquartile range (IQR) and were compared by the Mann–Whitney *U*-test. Pearson correlation analysis was employed to show the association of potential risk factors with neurocognition and the Ads of nine cognitive domains with MoCA-BC after data conversion in different grades of DAI patients, and presented in the form of a forestplot.

## Results

### Demographic and clinical characteristics of subjects

A total of 79 DAI patients diagnosed by clinical MRI within 30 days after injury were enrolled during the study period. At 6 months after injury, 54 patients met all the criteria for inclusion, eight patients were excluded due to the exclusion criteria, and a total of 46 DAI patients with favorable outcome were analyzed. Among them, eight had normal cognitive function (MoCA-BC ≥ 26) and 38 had cognitive impairment (MoCA-BC < 26). Comparing the two groups of DAI patients with 6-month favorable outcome with or without cognitive impairment, it was found that there were significant statistical differences in patients’ age, years of education, and time to return of consciousness (*p* = 0.003, *p* = 0.009, and *p* < 0.001). But surprisingly, there were no significant statistical differences in pupillary light reflex, admission GCS, Marshall CT score, and DAI grade (Table 2).

**TABLE 2:** Demographic and clinical characteristics of eligible patients with or without cognitive impairment.

Variables	MoCA-BC		*P*-value
	≥26 (*n* = 8)	<26 (*n* = 38)	
Male, *n* (%)	7 (87.5)	22 (57.9)	0.226
Age (years), median (IQR)	29.5 (25.8–35.8)	61 (47–65)	0.003*
**Cause of trauma, *n* (%)**			
Road traffic accident	5 (62.5)	24 (63.2)	>0.999
Fall	1 (12.5)	13 (34.2)	0.403
Others	2 (25)	1 (2.6)	0.074
**Pupillary light reflex, *n* (%)**			
None pupillary light reflex	0 (0)	7 (18.4)	0.325
Unilateral pupillary light reflex	1 (12.5)	8 (21.1)	>0.999
Bilateral pupillary light reflex	7 (87.5)	23 (60.5)	0.230
GCS, median (IQR)	13 (9–14.3)	7 (6–11)	0.051
Marshall CT score, median (IQR)	2.5 (2–3.5)	4 (3–5)	0.133
Education (years) (IQR)	12 (11.5–14.5)	6 (6–9)	0.009*
Return of consciousness (days) (IQR)	2 (1–5.8)	19 (9.3–28.3)	<0.001*
DAI grade 1 *n* (%)	6 (75)	18 (47.4)	0.247
DAI grade 2 *n* (%)	2 (25)	10 (26.3)	>0.999
DAI grade 3 *n* (%)	0 (0)	10 (26.3)	0.171

MoCA-BC, the Chinese version of the Montreal Cognitive Assessment Basic; GCS, Glasgow Coma Scale; DAI, Diffuse axonal injury; IQR, 25th percentile–75th percentile. *Represents a significant difference between the two groups.

### Relationship between risk factors and MoCA-BC scores

**FIGURE 2:**
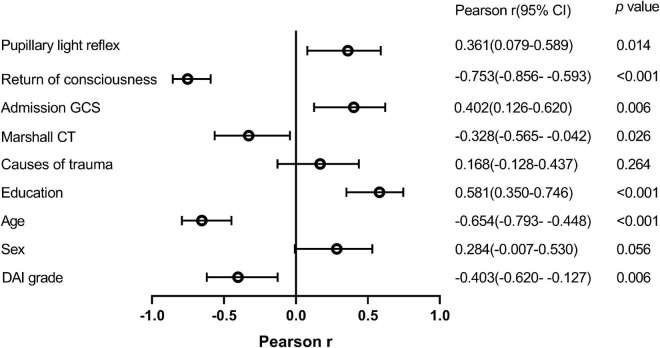
Relationship between potential risk factors and MoCA-BC scores. MoCA-BC, the Chinese version of the Montreal Cognitive Assessment Basic; GCS, Glasgow Coma Scale.

Pearson correlation analysis was employed to define the relationship of potential risk factors with the scores of MoCA-BC (Figure 2). The following four factors were found to be negatively correlated with the MoCA-BC scores: Return of consciousness (*r* = −0.753, *p* < 0.001), Marshall CT (*r* = −0.328, *p* = 0.026), age (*r* = −0.654, *p* < 0.001), and DAI grade (*r* = −0.403, *p* = 0.006). The MoCA-BC scores were positively correlated with pupillary light reflex (*r* = 0.361, *p* = 0.014), admission GCS (*r* = 0.402, *p* = 0.006) and years of education (*r* = 0.581, *p* < 0.001), Non-significant correlations were observed between sex, causes of trauma and the MoCA-BC scores.

### Cognitive impairment in different grades of DAI patients

**FIGURE 3:**
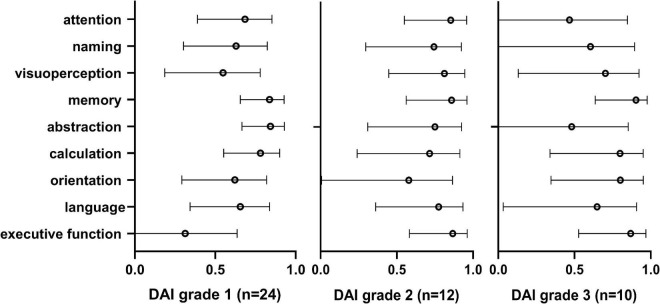
Pearson correlation analysis was performed between the Ads of nine cognitive domains and MoCA-BC after data conversion in different grades of DAI patients. MoCA-BC, the Chinese version of the Montreal Cognitive Assessment Basic; DAI, Diffuse axonal injury.

Moreover, to identify the association of cognitive impairment and each cognitive domain, Pearson correlation analysis was performed between the Ads of nine cognitive domains and MoCA-BC after data conversion in different grades of DAI patients (Figure 3). In patients with DAI grade 1, the Ads of memory (*r* = 0.838, *p* < 0.001), abstraction (*r* = 0.843, *p* < 0.001), and calculation (*r* = 0.782, *p* < 0.001) were most related to the Ads of MoCA-BC. The Ads of nine cognitive domains and MoCA-BC were all proved to be correlated, among patients with DAI grade 2. However, in the DAI grade 3 patients, the highest correlation with the Ads of MoCA-BC were the Ads of memory (*r* = 0.904, *p* < 0.001), calculation (*r* = 0.799, *p* = 0.006), orientation (*r* = 0.801, *p* = 0.005), and executive function (*r* = 0.869, *p* = 0.001). The whole correlation analysis results can be found in Supplementary Table 1.

## Discussion

This retrospective study indicated that 82.6% (38/46) of DAI patients with 6-month GOSE ≥5 were still plagued by cognitive impairment, and the major domains of cognitive impairment caused by different grades of DAI were also distinctive.

More than 50 million people are suffering from TBI each year worldwide, and most TBI patients are diagnosed by clinical MRI with some degree of DAI, especially severe TBI among them (Jiang et al., 2019; Lammy, 2020). DAI manifests in the form of focal axonal shear injuries and axonal breakage, so patients with DAI are more likely to have long-term sequelae or serious neurological deficits, even death (Lang et al., 2021; Palmieri et al., 2021).

GOSE or GOS are usually used by neurosurgeons to evaluate the prognosis of patients with DAI, but these assessment methods are not detailed and comprehensive, because previous studies have found that the rate of cognitive impairment is much higher than that of unfavorable outcome (Humble et al., 2018; Lohani et al., 2020; Macruz et al., 2022; RaukolaLindblom et al., 2022). In our study, MoCA-BC was performed to evaluate the cognitive function of 46 patients with DAI who were classified as favorable outcome, and 38 patients were found to have some degree of cognitive impairment. By comparing the two groups of patients with or without cognitive impairment in the present study, it was concluded that there were significant differences in age, education and return of consciousness, but there were no significant differences in admission GCS, Marshall CT score, pupillary light reflex and DAI grades.

In (Macruz et al., 2022) article, admission GCS and Marshall CT score had significant impact on cognitive function. Outcome was better in patients with DAI grade 1 and DAI grade 2 than in patients with DAI grade 3 (Skandsen et al., 2010; Moe et al., 2020). Have confirmed that age, GCS score, pupillary dilatation, traumatic axonal injury (TAI) grade on clinical MRI were significant predictors for poor outcome. Some of the results in our research were not consistent with the recent literature, which may be due to the deviation caused by the insufficient sample size.

Followed the long-term functional outcome of 134 patients with DAI and found independent prognostic factors were age, return of consciousness ≤7 days, pupillary reaction and DAI grade (van Eijck et al., 2018). Education level has been considered to be the strongest non-cognitive factor influencing performance on the MoCA (Chen et al., 2016). Analysis of 228 patients with TBI showed that education status was correlated with MoCA scores: Those patients with higher level of education had significant association with higher MoCA scores (*p* = 0.012) (Panwar et al., 2019). Marshall CT score, pupillary light reflex, duration of coma and admission GCS were predictors of clinical outcome in DAI (Palmieri et al., 2021; Xie et al., 2021; Chen et al., 2022). In the present study we also confirmed that return of consciousness, Marshall CT score, age and DAI grade have a negative correlation with cognitive function in DAI patients with 6-month GOSE 5–8. The more sensitive the pupillary light reflex, the higher the admission GCS and the longer the education, the better the long-term cognitive function of DAI patients. The correlation of these risk factors was basically consistent for the cognitive function of TBI patients and DAI patients with favorable outcome.

Severe cognitive impairment and physical disability were caused by lateral hypothalamus and medial hypothalamus damage during DAI (Wang et al., 2021). HVLT, TMT, and Rey–Osterrieth Complex Figure test were performed by Macruz et al. (2022) to reveal the impairment and recovery of episodic verbal memory, attention, and executive function of DAI patients. Further, scholars found that cognitive impairment was associated with brain tissue lesions displayed on MRI (Yue et al., 2020). Twenty-four patients with moderate or severe DAI were evaluated at 2, 6, and 12 months post-injury (Stewan et al., 2018) found out that microhaemorrhage load (MHL) was correlated only with white matter volume (WMV) reduction, executive function and episodic verbal memory were not correlated with MHL, but were, in part, correlated with WMV and total brain volume reduction. Not only severe TBI, but repetitive mild brain injury impair cognitive abilities and increase risk of neurodegenerative disorders in humans (Sai et al., 2020). So far, the persistent cognitive sequelae of patients suffering from DAI have been identified by the current studies, but the specificity of the impairment of nine cognitive domains caused by different grades of DAI have not been noticed by scholars. In our study, according to the diagnostic results of clinical MRI, DAI was divided into three grades for hierarchical interpretation. Finally, it was found that different degrees of DAI lead to significant differences in the domains of cognitive impairment. The decline of cognitive function in patients with DAI grade 1 was mainly caused by dysfunction in the three cognitive domains of memory, abstraction and calculation. But for patients with DAI grade 2, after the damage of the important structure (corpus callosum) connecting the left and right sides of the brain (Shah et al., 2021; Lewis et al., 2022), the cognitive impairment was most widely distributed, and none of the nine cognitive domains was immune. Patients with DAI grade 3 suffered from brainstem (the bridge of morphological and functional connection between telencephalon, diencephalon, cerebellum, and spinal cord) (Henson et al., 2020; Paton, 2020) injury, showing cognitive impairment dominated by memory, calculation and orientation and executive function dysfunction.

There are several limitations in our present study. First, insufficient sample size may lead to deviation and compromise the statistical power. Second, taking 6-month GOSE score ≥5 as the inclusion criteria may exclude some DAI patients who are classified as unfavorable outcome but can cooperate with the cognitive function test, thus affecting the final cognitive domain analysis results. Third, MoCA-BC was translated according to Chinese culture and may not be suitable for people in other countries.

## Conclusion

Our current research has revealed that DAI patients with favorable outcome may still be plagued by cognitive impairment. Different grades of DAI cause different domains of cognitive impairment, which requires neurosurgeons to provide patients with targeted cognitive rehabilitation programs.

## Data availability statement

The original contributions presented in this study are included in the article/Supplementary material, further inquiries can be directed to the corresponding authors.

## Ethics statement

The studies involving human participants were reviewed and approved by the Institutional Review Board of Haining People’s Hospital. The patients/participants provided their written informed consent to participate in this study.

## Author contributions

WC, CY, SL, JS, GW, and RJ: conceptualization and writing – review and editing. HH, ZZ, WS, and RC: data curation. XH and LX: formal analysis. LX, KS, JS, WC, CY, and HH: investigation. WC, SL, RJ, and GW: methodology. RJ and GW: supervision. HH and ZZ: visualization. WC and CY: writing – original draft. All authors contributed to the article and approved the submitted version.
