# Supplementary material for: Cognitive impairment in diffuse axonal injury patients with favorable outcome
Source: Front Neurosci. 2023 Jan 25;17:1077858. doi: 10.3389/fnins.2023.1077858 (PMC9905128; doi:10.3389/fnins.2023.1077858)
Supplement: Supplementary file 1 [file Table_1.DOCX]

|  |  |  |  |  |  |  | MoCA-BC vs. executive function | MoCA-BC vs. language | MoCA-BC vs. orientation | MoCA-BC vs. calculation | MoCA-BC vs. abstraction | MoCA-BC vs. memory | MoCA-BC vs. visuoperception | MoCA-BC vs. naming | MoCA-BC vs. attention |
| --- | --- | --- | --- | --- | --- | --- | --- | --- | --- | --- | --- | --- | --- | --- | --- |
|  |  |  |  |  |  | r | 0.3135 | 0.6561 | 0.6222 | 0.7815 | 0.8432 | 0.8376 | 0.5483 | 0.6295 | 0.6852 |
|  |  |  |  |  |  | 95%CI | -0.1029 - 0.6364 | 0.3436-0.8378 | 0.2921-0.8198 | 0.5522-0.9009 | 0.6666-0.9302 | 0.6558-0.9276 | 0.1860-0.7793 | 0.3030-0.8237 | 0.3894-0.8528 |
|  |  |  |  |  |  | *p* | 0.1357 | 0.0005 | 0.0012 | <0.0001 | <0.0001 | <0.0001 | 0.0055 | 0.0010 | 0.0002 |

DAI grade 1

|  | MoCA-BC vs. executive function | MoCA-BC vs. language | MoCA-BC vs. orientation | MoCA-BC vs. calculation | MoCA-BC vs. abstraction | MoCA-BC vs. memory | MoCA-BC vs. visuoperception | MoCA-BC vs. naming | MoCA-BC vs. attention |
| --- | --- | --- | --- | --- | --- | --- | --- | --- | --- |
| r | 0.8667 | 0.7744 | 0.5782 | 0.7150 | 0.7499 | 0.8588 | 0.8125 | 0.7435 | 0.8539 |
| 95%CI | 0.5826- 0.9621 | 0.3610- 0.9335 | 0.006473- 0.8651 | 0.2393- 0.9139 | 0.3090- 0.9255 | 0.5618- 0.9597 | 0.4472- 0.9455 | 0.2959- 0.9234 | 0.5490- 0.9582 |
| *p* | 0.0003 | 0.0031 | 0.0489 | 0.0090 | 0.0050 | 0.0003 | 0.0013 | 0.0056 | 0.0004 |

DAI grade 2

|  | MoCA-BC vs. executive function | MoCA-BC vs. language | MoCA-BC vs. orientation | MoCA-BC vs. calculation | MoCA-BC vs. abstraction | MoCA-BC vs. memory | MoCA-BC vs. visuoperception | MoCA-BC vs. naming | MoCA-BC vs. attention |
| --- | --- | --- | --- | --- | --- | --- | --- | --- | --- |
| r | 0.8688 | 0.6486 | 0.8012 | 0.7985 | 0.4813 | 0.9038 | 0.7026 | 0.6047 | 0.4678 |
| 95%CI | 0.5281- 0.9686 | 0.03202- 0.9076 | 0.3463- 0.9511 | 0.3396- 0.9503 | -0.2128- 0.8526 | 0.6362- 0.9773 | 0.1309- 0.9236 | -0.04023- 0.8940 | -0.2294- 0.8477 |
| *p* | 0.0011 | 0.0425 | 0.0053 | 0.0056 | 0.1590 | 0.0003 | 0.0235 | 0.0640 | 0.1727 |

DAI grade 3
